# Identification of novel light-induced genes in the suprachiasmatic nucleus

**DOI:** 10.1186/1471-2202-8-98

**Published:** 2007-11-19

**Authors:** Veronica M Porterfield, Helen Piontkivska, Eric M Mintz

**Affiliations:** 1 School of Biomedical Sciences, Kent State University, Kent, OH 44242 USA; 2 Department of Biological Sciences, Kent State University, Kent, OH 44242 USA

## Abstract

**Background:**

The transmission of information about the photic environment to the circadian clock involves a complex array of neurotransmitters, receptors, and second messenger systems. Exposure of an animal to light during the subjective night initiates rapid transcription of a number of immediate-early genes in the suprachiasmatic nucleus of the hypothalamus. Some of these genes have known roles in entraining the circadian clock, while others have unknown functions. Using laser capture microscopy, microarray analysis, and quantitative real-time PCR, we performed a comprehensive screen for changes in gene expression immediately following a 30 minute light pulse in suprachiasmatic nucleus of mice.

**Results:**

The results of the microarray screen successfully identified previously known light-induced genes as well as several novel genes that may be important in the circadian clock. Newly identified light-induced genes include early growth response 2, proviral integration site 3, growth-arrest and DNA-damage-inducible 45 beta, and TCDD-inducible poly(ADP-ribose) polymerase. Comparative analysis of promoter sequences revealed the presence of evolutionarily conserved CRE and associated TATA box elements in most of the light-induced genes, while other core clock genes generally lack this combination of promoter elements.

**Conclusion:**

The photic signalling cascade in the suprachiasmatic nucleus activates an array of immediate-early genes, most of which have unknown functions in the circadian clock. Detected evolutionary conservation of CRE and TATA box elements in promoters of light-induced genes suggest that the functional role of these elements has likely remained the same over evolutionary time across mammalian orders.

## Background

Circadian rhythms in mammals are driven by a clock located in the suprachiasmatic nucleus of the hypothalamus (SCN) [1]. The SCN is directly innervated by the retinas and photic information is transmitted to the SCN via a NMDA and pituitary adenylate cyclase activating peptide-dependent mechanism [2]. Photic input to the circadian clock has differential effects on circadian rhythms depending on the timing of exposure to light. Light presented in the early night delays the phase of the clock, light during the late night advances the clock, and light presented during the subjective day has little or no effect on clock phase [3]. Photic signals during the subjective night activate a MAP kinase signaling pathway leading to increased transcription of several immediate-early genes [4] and the core clock gene *Per1 *[2].

The roles of light-induced genes in the circadian clock mechanism, other than the period genes, are not well understood. For example, the best characterized of these genes are *Fos*, *Egr1*, and *Nr4a1 (nur77*). The expression of c-fos protein is commonly used as a marker for light-like activation of neurons in the SCN. Fos knockout mice still show behavioral circadian rhythms as well as a phase response curve to light, but the amplitude of the circadian rhythm of activity and the phase response curve are attenuated [5]. The threshold for photic induction of c-fos is similar to the threshold for behavioral phase shifts, suggesting a common mechanism [6], however, expression of c-fos can be dissociated from behavioral shifts [7-9]. *Fos *appears to be a component of the circadian response to light, but not one that is absolutely necessary for phase shifts to occur, and the mechanism by which Fos protein is involved in photic signalling is unknown. In contrast, *Egr1 *and *Nr4a1 *knockout mice show normal entrainment patterns and no dysfunction in their response to light [10]. The expression of egr1 occurs over a broader area of the SCN than does c-fos [11], but the threshold for egr1 induction is lower than for phase shifts of behavioral rhythms or c-fos induction. These data suggest that *Egr1 *and *Nr4a1 *may not be directly involved in entrainment pathways, but the lower threshold for stimulation of expression suggests that egr1 may be involved in other SCN outputs, such as the one which regulates rhythmic melatonin secretion from the pineal gland, which has a lower light intensity threshold for photic regulation than behavioral phase shifts.

In order to better understand the molecular events governing the response of the SCN to photic input, we performed a microarray-based screen for genes rapidly induced by light, followed by a comparative evolutionary genomic analysis to identify common activation mechanisms among this gene population.

## Results

### Light-induced immediate-early genes in the SCN

**Figure 1:**
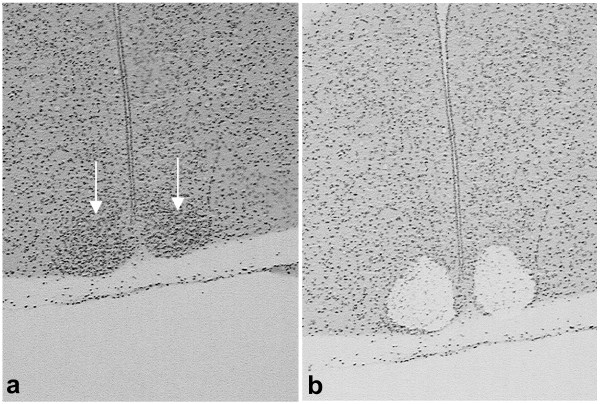
**Laser capture and gene expression in response to a light pulse**. (a) Twelve μm thick coronal section containing the SCN, indicated by arrows. Sections are stained with hemotoxylin. (b) The same section after capture, showing the removal of the paired SCN.

We used laser capture microscopy (Figure 1) to isolate SCN tissue from mice immediately following either a 30 minute high-intensity light pulse or a sham light pulse. The extent of the SCN was easily determined from the density of hemotoxylin-labeled nuclei. We examined a selected set of genes known to be markers of the SCN, but not the surrounding tissue, to confirm that we were measuring SCN gene expression. All 6 arrays displayed strong hybridization signals for the mRNA for vasoactive intestinal polypeptide (VIP), gastrin-releasing peptide, arginine vasopressin, calbindin 28 K, Clock, enkephalin, BMAL, and glutamic acid decarboxylase 67. VIP produced hybridization signals that were in the top 20 genes on 5 out of the 6 arrays (21^st ^on the 6^th ^array). In contrast, neural markers that are not expressed in the SCN, but are expressed in SCN-projecting neurons, received absent calls from all 6 arrays, including neuropeptide Y, preprohypocretin, tryptophan hydroxylase, and tyrosine hydroxylase.

**Figure 2:**
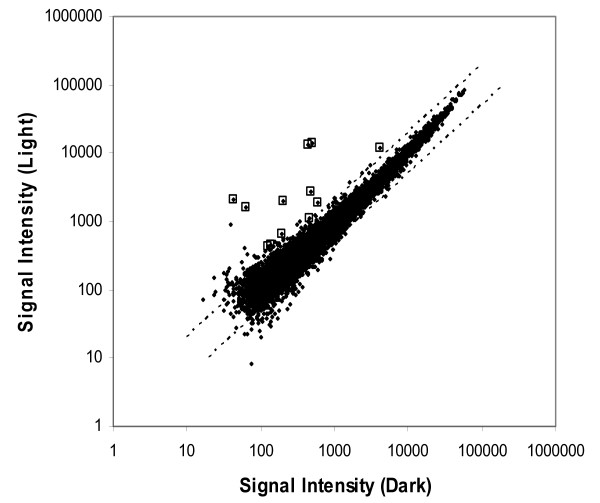
**Gene expression after light pulse vs. sham light pulse**. Summary comparison of gene expression after a light pulse vs. control dark pulse. Each point represents one gene that was present on at least half of the arrays. The boxes indicate data points that were subsequently confirmed as significantly different between conditions by qPCR.

**Figure 3:**
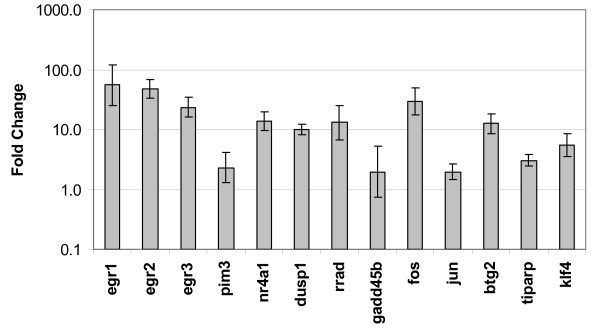
**Fold change of light-induced genes, measured by qPCR**. Fold change following a light pulse of genes significantly upregulated following the light pulse as compared to the sham light pulse, as measured by qPCR. Error bars represent 95% confidence intervals.

The 22,626 probe sets on the Affymetrix arrays were reduced to 10,340 candidate transcripts for analysis based on the criteria that each probe set was identified as being present using the default settings of GCOS 1.1 software on at least 3 of the 6 arrays. The results of these probe sets are plotted in Figure 2. Differences between light pulse and sham light pulse groups were assessed according to p-values generated using a Bayesian t-test calculated using the logarithms of the signal intensities for each probe set. A total of six genes were measured as being differentially expressed using our most stringent criteria, which was the application of a Bonferroni correction to the critical p-value for significance (p < 4.84E-06). A second group of 9 genes was identified as meeting a lesser criteria of p < 0.001. All 15 genes of these genes were then analyzed for differences in expression using quantitative real-time PCR (qPCR). All 6 of the genes in the first group were confirmed as having a more than 2-fold increase in expression following the light pulse, while 6 of the 9 genes in the second group were confirmed. In addition, we tested the fold-change of 3 additional genes that had not been identified in our microarray analysis but had previously been shown to be induced by a light pulse (*Egr3*, *Per1*, and *Per2*) [12,13]. Of this group, *Egr3 *showed a significant increase in expression following the light pulse but no significant difference in *Per1 *or *Per2 *expression was detected. The identities of these genes and their fold-changes as calculated using the microarrays and qPCR are shown in Table 1 and Figure 3. The correlation coefficient between fold changes determined by microarray analysis and by real-time PCR was 0.87 (Spearman's rank-correlation, *p *= 0.00003). There were no transcripts that showed a significant decrease in expression following the light pulse.

**Table 1:** Fold change in expression following a light pulse

Gene Symbol	Gene Title	Entrez Gene ID	Fold-Array*	p-value**
Egr1	early growth response 1	13653	27.7	3.45E-13
Nr4a1	nuclear receptor subfamily 4, group A, member 1	15370	10.2	2.13E-09
Egr2	early growth response 2	13654	47.3	4.19E-08
Dusp1	dual specificity phosphatase 1	19252	5.8	6.18E-08
Rrad	Ras-related associated with diabetes	56437	26.2	7.89E-08
Pim3	proviral integration site 3	223775	2.9	1.32E-06
Klf4	Kruppel-like factor 4 (gut)	16600	3.4	2.07E-04
Fos	FBJ osteosarcoma oncogene	14281	29.3	2.18E-04
Gadd45b	growth arrest and DNA-damage-inducible 45 beta	17873	3.2	2.58E-04
Btg2	B-cell translocation gene 2, anti-proliferative	12227	3.3	5.84E-04
Tiparp	TCDD-inducible poly(ADP-ribose) polymerase	99929	2.4	6.02E-04
Jun	Jun oncogene	16476	3.5	6.99E-04

* Fold-array indicates the fold-difference in gene expression following a light pulse as compared to a sham light pulse. ** Bayesian t-test using Cyber-T software (see Methods section for details)

The failure to detect an increase in *Per1 *expression was unexpected, as some previous reports detected an increase in *Per1 *mRNA expression within 10 minutes of the onset of a nocturnal light pulse [14]. However, we hypothesized that the increase in *Per1 *might be occurring later in our study. Therefore, we harvested SCN tissue from mice one hour after the start of a 30 minute light pulse or sham light pulse, 30 minutes later than in our initial study. qPCR assays of these captures revealed a significant increase in both *Per1 *(7.0× over dark pulse, 95% confidence interval of 2.8 – 17.8, n = 4 per group) and *Per2 *(1.9× over dark pulse, 95% confidence interval of 1.1 – 3.4, n = 4 per group) expression after the light pulse as compared to the sham light pulse.

### Analysis of promoter regions of light-induced genes

**Figure 4:**
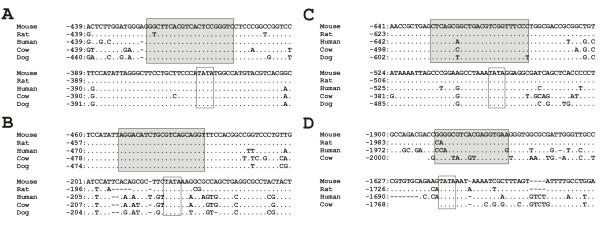
**Conserved promoter elements in light-induced genes**. Conserved CRE and TATA-box elements in promoter regions of four immediate-early genes in five mammalian genomes: (A) *Egr1*, (B) *Fos*, (C) *Per1 *and (D) *Klf4*. Translational start codon ATG begins at +1. Alignment gaps are shown as dashes (-), dots (.) indicate identity to the first sequence. For Klf4, genomic sequence of dog was not available. Predicted conserved CRE elements are outlined with shaded boxes; putative TATA-boxes are shown with white boxes.

We reasoned that if these light-induced genes were important to the basic mechanism of photic signalling to the SCN and were activated by a common mechanism, they may share common regulatory sequences. Ca^2+^/cAMP response elements (CRE) have been shown to play a critical role in circadian rhythmicity and the photic entrainment system [15,16]. We therefore took a comparative evolutionary genomic approach to ask whether all of the light-inducible genes in the SCN that we detected contain conserved CRE elements in their promoters. We found at least one highly conserved CRE element within 2 kb upstream of the start codon in 12 of the 13 genes induced by light immediately following the light pulse (Figure 4, Table 2 Additional file 1). The only gene lacking a conserved CRE element within 2 kb of the start codon was *Nr4a1*. Most of these CRE elements were highly conserved not only at the nucleotide sequence level but also in their relative positions within the promoter region in the five mammalian species examined (mouse, rat, human, cow and dog), suggesting that these elements are important to coordinate the regulation of this group of genes. Previous studies have also demonstrated that cAMP-dependent activation of transcription by p-CREB largely depends on the presence of TATA elements downstream of the CRE element [17]. We found that 8 out of the 12 genes with CRE elements also possess TATA elements in proximity to the CRE element, including the 4 genes that showed the largest increases in expression following the light pulse. By these same criteria, *Per1 *has both a conserved CRE element and a conserved TATA box, while *Per2 *has neither. In contrast, in 8 other clock genes (Table 3) that were not upregulated 30 minutes after the light pulse onset in this study, only one (*Csnk1d*) had both a conserved CRE element and a nearby TATA box. This difference (12/13 vs. 1/8) is statistically significant (Fisher's Exact Test, p < 0.001).

**Table 2:** Conservation of CRE and TATA elements in light-induced genes

**Ensembl gene ID**	**Gene name**	Number of conserved CRE elements*	Genomic positions of CRE elements**	**Genomic positions of TATA boxes**
ENSMUSG00000020423	Btg2	2	-277 to -257 (3) -259 to -239	No TATA
ENSMUSG00000024190c	Dusp1	2	-349 to -329 -301 to -281 (4)	-180 to -175***
ENSMUSG00000038418	Egr1	3	-925 to -905 -424 to -404 -355 to -335	-363 to -360 -686 to -682
ENSMUSG00000037868	Egr2	1	-414 to -394	-302 to -298
ENSMUSG00000033730	Egr3	2	-645 to -625 (2) -592 to -572	-599 to -596
ENSMUSG00000021250	Fos	3	-500 to -480 -452 to -432 (2) -225 to -205 (2)	-183 to -178
ENSMUSG00000015312	Gadd45b	1	-925 to -905	No TATA
ENSMUSG00000052684	Jun	2	-1261 to -1241 -1002 to -982	-505 to- 502*****
ENSMUSG00000003032	Klf4	2	-1023 to - 1003 -986 to -966	-778 to -774
ENSMUSG00000023034	Nr4a1	N/A****	N/A	No TATA
ENSMUSG00000020893	Per1	1	-355 to -335 (2)	-500 to -497
ENSMUSG00000055866	Per2	N/A	N/A	No TATA
ENSMUSG00000035828	Pim3	1	-686 to -666 (2)	No TATA
ENSMUSG00000031880c	Rrad	1	-522 to -502	No TATA
ENSMUSG00000034640	Tiparp	1	-1863 to - 1843	-1615 to -1610

* We defined CRE element as being evolutionary conserved if it was present in at least 3 out of 5 mammalian genomes (Mouse, Rat, Human, Cow and Dog). ** Number in parenthesis indicates how many overlapping CRE elements were identified in the promoter sequence, however, coordinates of only the first of the overlapping elements are given here. *** Mouse and rat promoters have non-canonical CATAAAA variants instead of canonical TATAA in human, cow and dog. However, this non-canonical variant is known to function in a TATA box-like fashion [56,57]. **** N/A = not applicable, because in Nr4a1 and Per2, no conserved CREs were detected within 2 kb of the promoter sequence. ***** In human sequence TATA box was identified within 300 bp downstream of conserved CRE; however, it was not present in other genomes. See additional file 1 for additional notes for this table.

**Table 3:** CRE and TATA elements in selected clock genes

**Ensembl gene ID**	**Gene name**	Number of conserved CRE elements within the promoter sequence *	**Genomic positions of upstream CRE elements in mouse promoter relative to start codon (ATG starts at position +1)**	**Genomic positions of TATA boxes if any**
ENSMUSG00000020038	Cry1	1	-600 to -580	No TATA
ENSMUSG00000068742	Cry2	1	-262 to -242	No TATA
ENSMUSG00000029238	Clock	No CRE ***	N/A	-472 to -469 ****
ENSMUSG00000055116	Bmal1	No CRE	N/A	No TATA
ENSMUSG00000020889	Rev-erb-alpha (Nr1d1)	No CRE	N/A	No TATA
ENSMUSG00000032238	Ror-alpha (Rora)	No CRE	N/A	No TATA
ENSMUSG00000022433	Casein kinase 1 epsilon (Csnk1e)	No CRE	N/A	No TATA
ENSMUSG00000025162	Casein kinase 1 delta (Csnk1d)	1	-330 to -310 (4) **	-570 to -567

* We defined CRE element as being evolutionary conserved if it was present in at least 3 out of 5 mammalian genomes (Mouse, Rat, Human, Cow and Dog). ** Number in parenthesis indicates how many overlapping CRE elements were identified in the promoter sequence, however, coordinates of only the first of the overlapping elements are given here. *** Only mouse and human promoters had predicted CREs; however, these CREs were located in different positions and therefore we did not consider them as being evolutionary conserved. **** Multiple TATA boxes were identified in each species; however, not all of them were evolutionary conserved in all sequences. TATA box shown was shared between four out of five species because dog genomic sequence was not available in that region.

## Discussion

The results of this study identify a total of 13 transcripts that show significant increases in expression 30 minutes after the onset of a light pulse during the early night. It is important to note that this study examined gene expression in only the earliest moments of the response to photic stimulation. It is likely that repeating this study at later time points would reveal additional light-induced genes.

Four of the differentially expressed transcripts identified in the microarray analysis have not previously been identified as being induced by light: *Egr2*, *Pim3*, *Gadd45b*, and *Tiparp*. *Egr2 *is a zinc-finger transcription factor of the same family as *Egr1 *and *Egr3*. There is insufficient information available to generate a clear hypothesis about the function of Egr genes in the SCN. *Pim3 *is a serine/threonine protein kinase that has anti-apoptotic functions in a variety of tissues [18,19], but its role in neurons has not been investigated. In contrast, the known functions of *Gadd45b *and *Tiparp *suggest potential connections to SCN function. In beta cells, Gadd45b inhibits apoptosis as well as JNK and ERK activation [20]. Gadd45b has similar functions in hematopoietic cells [21]. *Gadd45b *is upregulated in the hippocampus in response to electroconvulsive shock, consistent with a neuroprotective function [22]. If Gadd45b acts to inhibit ERK activation in the SCN, then that would be consistent with the idea that some of these light-induced genes act to downregulate the sensitivity of the SCN to subsequent stimuli, and possibly contribute to the SCN's strong resistance to excitotoxicity [23].

*Tiparp *encodes TCDD-inducible poly [ADP-ribose] polymerase and is activated through an aryl hydrocarbon receptor-dependent pathway [24]. Activation of this pathway by tryptophan photoproducts alters the expression of clock genes and inhibits glutamate-induced phase shifts in SCN 2.2 cells [25]. The aryl hydrocarbon receptor shares significant structural similarity with Bmal1, a core clock gene, and may interact directly with Bmal1, providing a potential substrate for altering clock function [26]. The induction of *Tiparp *provides another potential mechanism for altering clock function, although the targets for ribosylation in the SCN are unknown.

Three genes identified in our study (*Rrad*, *Btg2*, *Klf4*) were recently reported as light-induced in a study using high-coverage expression profiling [27], and another gene, *Dusp1*, was also recently characterized as light-induced [28]. *Dusp1 *encodes MAP kinase phosphatase 1 (MKP1), and is now thought to play a role in the termination of the photic signalling cascade. One of the critical steps in the response of the SCN to photic signaling is the phosphorylation of the extracellular signal regulated kinases (ERK), leading to phosphorylation of CREB and changes in transcription [29,30]. An increase in functional MKP1 would reduce the sensitivity of the SCN to photic stimuli, and shut down the transcriptional mechanisms turned on by light. It also might help explain the extraordinary resistance of SCN cells to excitotoxicity [23], by downregulating the responses of SCN cells during periods of extended stimulation.

*Rrad*, *Btg2*, and *Klf4 *have not been examined in the context of circadian clock function. However, given what is known about the role of *Rrad *in other systems, increased Rrad protein would also serve to downregulate the response of SCN cells to subsequent stimulation. *Rrad *encodes a small GTPase that binds to calmodulin and CaM Kinase II [31]. Increased Rrad expression is associated with the removal of voltage-gated calcium channels from the plasma membrane [32], which would reduce the response of SCN neurons to excitatory stimuli. In addition, CaM Kinase II has already been shown to play a role in the regulation of light-induced phase delays, through actions leading to the transcription of *Per1 *[33,34]. Consistent with this theme of downregulating cellular responses to stimuli, *Btg2 *and *Klf4 *both have anti-apoptotic functions in other cellular systems [35,36], but their role in the SCN remains unknown.

The remaining five genes are well known light-induced genes: *Egr1*, *Egr3*, *Fos*, *Nr4a1*, and *Jun*. Investigations into the function of these genes in the response of the SCN to photic stimuli have been extremely limited. Mice lacking functional copies of *Egr1 *and *Nr4a1 *(*nur77*) do not show altered circadian rhythms, suggesting that these genes do not play an important role in entrainment [10], although there could be compensatory mechanisms in the knockout mice that mask the role of these genes. In contrast, mice lacking *Fos *show an attenuated phase response curve to light [5], suggesting that this gene is involved in the signal transduction mechanism for conveying photic information to the molecular clock mechanism. The critical factor in the response of the circadian clock to light is the increase in expression of period genes, through a mechanism that does not appear to require Fos. One possibility is that mechanisms within the cell that are activated by Fos protein are important for the communication of information from retinorecipient cells to other parts of the SCN. Another piece of evidence implicating both *Fos *and *Jun *in entrainment is that microinjection of antisense oligonucleotides for these transcripts into the brain inhibits the phase shifting effects of light [37]. In addition, the behavioral phase shifts are highly correlated with changes in *Fos *expression, but not with *Egr1 *or *Nr4a1 *expression [38]. There have been no functional studies regarding the role of *Egr3 *in the circadian clock.

We found that nearly all of the light-induced genes identified in this study had evolutionary conserved CRE elements in their promoter regions, with the strongest light inducible responses coming from the combination of CRE elements and associated TATA boxes. On the other hand, core clock components that are not induced by light lack this combination of promoter elements, with one exception (casein kinase 1 delta). The slower, longer-lasting increase in *Per2 *relative to the other induced genes has been described elsewhere [39]. The loss of a conserved CRE element from the *Per2 *promoter may be a component of its functional divergence from *Per1*. It is likely that while the CRE element helps drive transcription of light-induced genes, there are other important regulatory elements responsible for controlling the time course of expression in response to activation by p-CREB. However, these data strongly suggest that the presence of the CRE element drives transcription of a coordinated array of immediate-early genes in the SCN in response to photic stimulation. Further evidence of this is the finding that CREB specifically binds to the CRE sequences in the *Dusp1 *promoter [28]. Observed evolutionary conservation of CRE elements among mammalian genomes that diverged at least 90 million years ago (MYA), such as primate-cattle divergence at about 90–98 MYA [40], and primate-rodent divergence at about 90 MYA [41], indicates that the functional role of these elements in regulation of clock genes has likely remained the same in different mammals. Although this kind of *in silico *analysis requires further experimental evidence, and while the role of many light-induced genes in the SCN remains unknown, these data support the idea that the response of the SCN to photic input is not limited to pathways involved directly in entrainment.

## Conclusion

The results from this study demonstrate the existence of previously unknown light-induced immediate-early genes in the SCN. Several of these genes have been previously shown to be involved in a reduction of cellular activity and/or the prevention of apoptosis. These data suggest that in addition to the responding to light by shifting the timing of the circadian clock, mechanisms exist to reduce the long-term sensitivity of the SCN to light during nocturnal light exposure. Most light-induced genes have evolutionary conserved CRE elements in their promoter regions, supporting a common mechanism for a coordinated transcriptional response to photic input. Further investigation into the functional role of light-induced genes may yield new insight into the mechanisms of circadian clock function.

## Methods

### Animals

Adult male C57BL/6 mice were individually housed in a 14:10 light/dark cycle in their experimental room and cage for at least two weeks prior to the experiment. They were then exposed to a 30 minute light pulse (2400lux) or a dark pulse (sham) starting one hour after lights off. Previous research has shown that this duration and intensity of light is well below the threshold for induction of retinal apoptisis [42]. Immediately after treatment, mice were euthanized via cervical dislocation. Dark pulse mice were euthanized under dim red light, and both light and dark pulse animals' eyes were dissected out immediately to avoid excess exposure to light. The mice were then decapitated and the brains were quickly removed and frozen in isopentane cooled in dry ice. Brains were stored at -70°C until ready for further processing. Animals were handled in accordance with the guidelines of the PHS Guide to the Care and Use of Laboratory Animals and all NIH regulations.

### Laser Capture Microscopy/RNA purification

Brains were cut into 12 μm thick sections on a cryostat and directly mounted onto glass slides. Sections were stained using a quick protocol to allow for visual identification of the suprachiasmatic nucleus. First the sections were fixed in a 75% EtOH solution for 30 seconds, rinsed in water to remove excess EtOH from the slide, and then immersed in Hemotoxylin for 90 seconds. Slides were then washed in molecular biology grade water. The slides then were taken through an alcohol dehydration series of 75%, 95% and 100% EtOH for 30 seconds each, followed by immersion in xylenes for 5 minutes. The slides were removed from the xylene, and once the remaining xylenes had evaporated the slides were placed into a laser capture microscope (Arcturus) and the SCN was identified and captured into CapSure^® ^HS LCM Caps (Molecular Devices).

For microarrays and validation of microarrays by quantitative real-time PCR (qPCR), 6 consecutive SCN sections were captured from each mouse. The specificity of each capture for SCN tissue was confirmed after capture by examination of both pre and post-capture images of the tissue. While it is possible that a few cells were technically extra-SCN from any given sample, we estimate that this would comprise less than 1% of the total captured material. Samples from three mice were then pooled together within the same treatment, so in total there were 18 SCN sections pooled together. For the *Per1 *and *Per2 *time course experiment, 6 consecutive SCN sections from each mouse were used but multiple mice were not pooled for analysis. The pooled samples were purified using an RNA purification kit (Picopure from Molecular Devices) including a DNase treatment. A 1 μl aliquot of each sample was removed and processed on an Agilent Bioanalyzer 2100 using the Agilent Lab-on-a-Chip Picochip RNA kit. Only samples with RNA integrity numbers about 6.8 were processed further. RNA was stored at -70°C until ready to proceed.

### Microarrays

For experiments involving microarrays, 1 ng of total RNA was processed through two rounds of linear amplification using RiboAmp HS kits (Molecular Devices). Amplified samples were labeled, fragmented, and hybridized to Affymetrix Mouse 430A 2.0 Genechips using the standard Affymetrix protocols according to the 2004 edition of the Affymetrix technical manual. Signal intensities for each gene were generated using the Microarray Suite 5.0 algorithm by Affymetrix GCOS software. In addition to the signal intensity, each gene was determined to be present, marginal, or absent using default software settings. Signal intensity and present/absent data were imported into Excel and filtered such that only genes that received a present call in at least 3 of the 6 arrays were included in the analysis. This reduced the total number of probe sets to be analyzed from 22,626 to 10,340. Signal intensities for the three light pulse and three dark pulse arrays were analyzed using Cyber-T software [43] using the default settings. This software generates p-values for each gene as a test of differences between light and dark pulse groups using a Bayesian t-test [44]. Two sets of candidate genes were selected based on the results of this test. The first set was characterized by Bayesian t-test p-values that were below the critical value for a Bonferroni multiple-comparison correction (p < 4.8E-06). The second set met a lesser criterion of p < 0.001. The false discovery rate was examined empirically by qPCR confirmation.

The data discussed in this publication have been deposited in NCBI's Gene Expression Omnibus (GEO, [45]) and are accessible through GEO Series accession number GSE6904.

### Real Time PCR

For experiments involving qPCR validation, Taqman 20× gene expression probes were ordered from Applied Biosystems for the genes shown to be significantly upregulated from the microarray results. qPCR was performed on an Applied Biosystems Prism 7000 sequence detection system. Purified Total RNA from above was reversed transcribed using Taqman Reverse Transcription reagents kit, using standard protocol with random hexamers. qPCR was conducted using Taqman Universal Master Mix on the experimental samples, with all samples being assayed in triplicate. Each plate run included a subset of primers for mouse gapdh as a control gene. Analysis of relative gene expression in real-time PCR experiments were performed using the 2^-ΔΔCT ^method[46].

### Analysis of evolutionary conservation of CRE and TATA elements in the promoter sequences

Genomic sequences of promoter sequences for five mammalian species were downloaded from Ensembl Genome Browser [47], release 42. The following genomes were used: NCBI build m36 assembly of mouse (*Mus musculus*) genome, NCBI 36 assembly of human (*Homo sapiens*) genome, whole genome shotgun (WGS) assembly CanFam2.0 of dog (*Canis familiaris*) genome, WGS preliminary assembly Btau_2.0 of cow (*Bos taurus*) genome, and RGSC 3.4 assembly of rat (*Rattus norvegicus*) genome. Promoters were defined as 2 kb upstream of the annotated translational start sites (ATG) of respective immediate-early and clock genes.

Putative CRE (cAMP-response) elements were identified using MatInspector [48,49] using vertebrate matrices of the Matrix Family Library Version 6.2 (October 2006) [50]. If multiple overlapping CREs were detected within 10 bp of each other, they were considered to form a single CRE. After CRE elements were identified in each sequence, a search for downstream TATA boxes was performed.

Multiple sequence alignments of the promoter sequences were constructed with the program BlastZ that is specifically fine-tuned to capture sequence similarity of large non-coding sequences such as those found in the promoter regions [51] using MultiPipMaker [52,53]. Putative CRE elements and TATA boxes from mouse promoters were mapped onto the alignments. If the same elements were detected in the homologous position in at least two out of four other mammalian genomes, such CREs and TATA boxes were considered evolutionary conserved and likely to be functional [52,54,55].

## Authors' contributions

VMP carried out the laser capture, microarray hybridization, and qPCR analysis and helped to design the study and draft the manuscript. HP carried out the comparative genomic analysis and participated in drafting the manuscript. EMM conceived of the study, performed the statistical analyses, and drafted the manuscript. All authors read and approved the final manuscript.

## Supplementary Material

Additional File 1 Supplementary notes for table 2. Additional information on the alignment of promoter sequences across species. Click here for file
