# Supplementary material for: Identification of novel light-induced genes in the suprachiasmatic nucleus
Source: BMC Neurosci. 2007 Nov 19;8:98. doi: 10.1186/1471-2202-8-98 (PMC2216081; doi:10.1186/1471-2202-8-98)
Supplement: Additional File 1 — Supplementary notes for table 2. Additional information on the alignment of promoter sequences across species. [file 1471-2202-8-98-S1.pdf]

Supplementary notes for Table 2.

ENSMUSG00000020423 (Btg2): -259 to -239 CRE is absent in human promoter; genomic sequence is not available in Dog.

ENSMUSG00000038418 (Egr1): -925 to -905 CRE is absent in human promoter.

ENSMUSG00000037868 (Egr2): only Mouse, Rat and Cow sequences can be aligned.

ENSMUSG00000003032 (Klf4): genomic sequence is not available in Dog.

ENSMUSG00000023034 (Nr4a1): only Mouse and Rat can be aligned. CRE is not identified in Rat; however, it is known to be not conserved in this gene (Uemura, Mizokami, and Chang 1995).

ENSMUSG00000055866 (Per2): only Mouse and Rat can be aligned. No CRE elements were predicted within 2kb upstream of ATG codon. However, CRE is known to be located further upstream in this gene (Travnickova-Bendova et al. 2002).

ENSMUSG00000035828 (Pim3): only Mouse and Rat sequences can be aligned.

ENSMUSG00000031880c (Rrad): genomic sequence is not available in Rat.

ENSMUSG00000034640 (Tiparp): genomic sequence is not available in Dog.

Reference:

Travnickova-Bendova, Z., N. Cermakian, S. M. Reppert, and P. Sassone-Corsi. 2002. Bimodal regulation of mPeriod promoters by CREB-dependent signaling and CLOCK/BMAL1 activity. Proc Natl Acad Sci U S A **99**:7728-7733.

Uemura, H., A. Mizokami, and C. Chang. 1995. Identification of a new enhancer in the promoter region of human TR3 orphan receptor gene. A member of steroid receptor superfamily. J Biol Chem **270**:5427-5433.
